# The Effect of Aquaporin 1-Inhibition on Vasculogenic Mimicry in Malignant Mesothelioma

**DOI:** 10.3390/ijms18112293

**Published:** 2017-11-01

**Authors:** Emily Pulford, James McEvoy, Ashleigh Hocking, Sarita Prabhakaran, Kim Griggs, Sonja Klebe

**Affiliations:** 1 Department of Anatomical Pathology, Flinders University, Adelaide 5000, SA, Australia; pulf0010@flinders.edu.au (E.P.); j.mcevoy@flinders.edu.au (J.M.); ash.hocking@flinders.edu.au (A.H.); sarita.prabhakaran@flinders.edu.au (S.P.); kim.griggs2@sa.gov.au (K.G.); 2 Department of Surgical Pathology, SA Pathology at Flinders Medical Centre, Adelaide 5001, SA, Australia

**Keywords:** malignant mesothelioma, vasculogenic mimicry, aquaporin 1, vascular endothelial growth factor A (VEGFA), angiogenesis

## Abstract

Malignant mesothelioma (MM) is an aggressive malignancy of the serosal membranes, with poor overall survival and quality of life. Limited targeted treatment strategies exist due to restricted knowledge of pathogenic pathways. Vasculogenic mimicry (VM) is a newly described phenomenon associated with increased aggressiveness in other malignancies, and has been characterized in MM. Normal mesothelium expresses aquaporin 1 (AQP1) and retained expression has been associated with improved survival in MM. AQP1 is expressed by normal vascular endothelium and is involved in mediating MM cell motility and proliferation. We investigated the role of AQP1 in VM, and its interaction with the pro-angiogenic factor vascular endothelial growth factor A (VEGFA), which is variably expressed in MM. Matrigel VM assays were performed using NCI-H226 and NCI-H28 MM cell lines and primary cells in hypoxia and normoxia. The synthetic blocker AqB050 and siRNA were used to inhibit AQP1, and bevacizumab was used to inhibit VEGF. Inhibition of AQP1 resulted in increased VEGFA secretion by MM cells and reduced VM in MM cell lines in hypoxia but not normoxia. No change in VM was seen in MM primary cells. Combined inhibition of AQP1 and VEGF had no effect on VM in normoxia. In a heterotopic xenograft mouse model, AqB050 treatment did not alter vessel formation. AQP1 may interact with VEGFA and play a role in VM, especially under hypoxic conditions, but the heterogeneity of MM cells may result in different dominant pathways between patients.

## 1. Introduction

Malignant mesothelioma is an aggressive malignancy of the serosal membranes, primarily attributable to asbestos exposure. Mean survival time is 12–18 months post-diagnosis. Response to current therapies such as chemotherapy, radiotherapy and surgery is poor. Limited targeted treatments exist for malignant mesothelioma (MM) due to the lack of definitive driver mutations, predictive markers and incomplete understanding of pathogenic pathways. Traditional pathways of angiogenesis have been suggested as targets for therapy due to adverse prognostication associated with increased serum and pleural effusion levels of the pro-angiogenic marker vascular endothelial growth factor A (VEGFA). VEGFA also contributes directly to MM tumour growth and progression [1,2]. Additionally, intratumoural microvessel density is associated with poor prognosis [3]. Clinical inhibition of this pathway utilises bevacizumab (Avastin™, Genentech, South San Francisco, CA, USA) and tyrosine kinase inhibitors of VEGF receptors; however, improvements to overall survival have been limited [4,5,6,7,8,9]. This indicates the requirement for identification of novel targets to improve treatment efficacy and quality of life.

Vasculogenic mimicry (VM) is a process whereby tumour cells form tumour cell-lined vasculature, meeting nutritional requirements at least in part, by adopting an endothelium-like phenotype [10]. This phenomenon has been associated with increased aggressiveness and decreased overall survival in a number of malignancies [11,12,13,14] and has recently been described in MM tumours [15]. Inhibition of VEGF has resulted in suppression of VM in some malignancies, including high-grade glial tumours [16] and osteosarcoma [17]. Schnegg et al. observed that VEGF silencing did not modulate VM in melanoma cell lines in vitro [18]. Conversely, increased VM was observed in an in vivo model where melanoma cell line WM1617 infected with VEGFA siRNA was introduced into SCID mice; however, use of other melanoma cell lines C8161 and A2058 did not result in any difference in VM in this in vivo model [18]. This suggests that the potential modulating activity of VEGF in VM may vary depending on cell type or cell line, or may possibly be related to activation of alternative pathways. The presence of multiple pathways involved in angiogenesis may be the reason why inhibition of VEGF in MM has not slowed disease progression significantly. The potential effects of VEGF inhibitors, or other molecules potentially involved in regulating VM, have not been studied in MM.

Aquaporin 1 (AQP1) is a water channel protein expressed on cell membranes throughout the body, including vascular endothelial cells [19]. AQP1 has been implicated in MM cell proliferation, adhesion and motility, as well as stabilisation of the cytoskeleton [20,21]. AQP1 activity has been implicated in modulation of pleural fluid volumes through osmotic equilibration [22]. This is potentially significant in MM, which commonly presents with large pleural effusions, which interfere with breathing and thus contribute to poor quality of life. Retained expression of AQP1 by immunohistochemistry in MM histology specimens has been associated with better overall survival, indicating a potential role in pathogenesis [23,24]. Furthermore, increased AQP1 protein and mRNA expression have been observed in epithelioid MM subtypes, which have a better prognosis when compared to biphasic and sarcomatoid subtypes [24,25]. Saadoun et al. explored the effect of AQP1 on tumour cell angiogenesis in a mouse model of melanoma, observing reduced microvessel density in AQP1-null mice, and detecting AQP1 expression in tumour vasculature in wild-type mice [26]. Additionally, they observed a reduction in vessel formation in AQP1-null mice compared to wildtype mice regardless of VEGF addition, using an in vivo Matrigel plug assay. Due to its established role in cell migration in MM, and in angiogenesis in other tumour types, we proposed that blockade of AQP1 may inhibit VM in MM cells.

The aim of this study was to investigate the potential role of AQP1 in VM of MM cell lines and primary cells under normoxic and hypoxic conditions to mimic the tumour microenvironment. Inhibition of AQP1 was achieved using a synthetic AQP1-specific pharmaceutical blocker (AqB050) and AQP1-specific siRNA. Inhibition of VEGFA was achieved by treatment with bevacizumab (Avastin™, Genentech, USA), a recombinant monoclonal antibody against VEGFA. We also investigated the effect of AQP1 blockade on vessel development in vivo in a heterotopic xenograft model of MM. We also aimed to determine the combined effect of AQP1 and VEGF inhibition on VM in MM cell lines and primary cells. This may provide insight into pathways of MM tumour progression and angiogenesis, and identify candidates for therapeutic intervention in MM patients.

## 2. Results

### 2.1. AQP1 Treatment Inhibits VM in Cell Lines under Hypoxic Conditions In Vitro, but not Does Inhibit VM under Normoxic Conditions in Cell Lines or Primary Cells, or Microvessel Formation In Vivo

**Figure 1:**
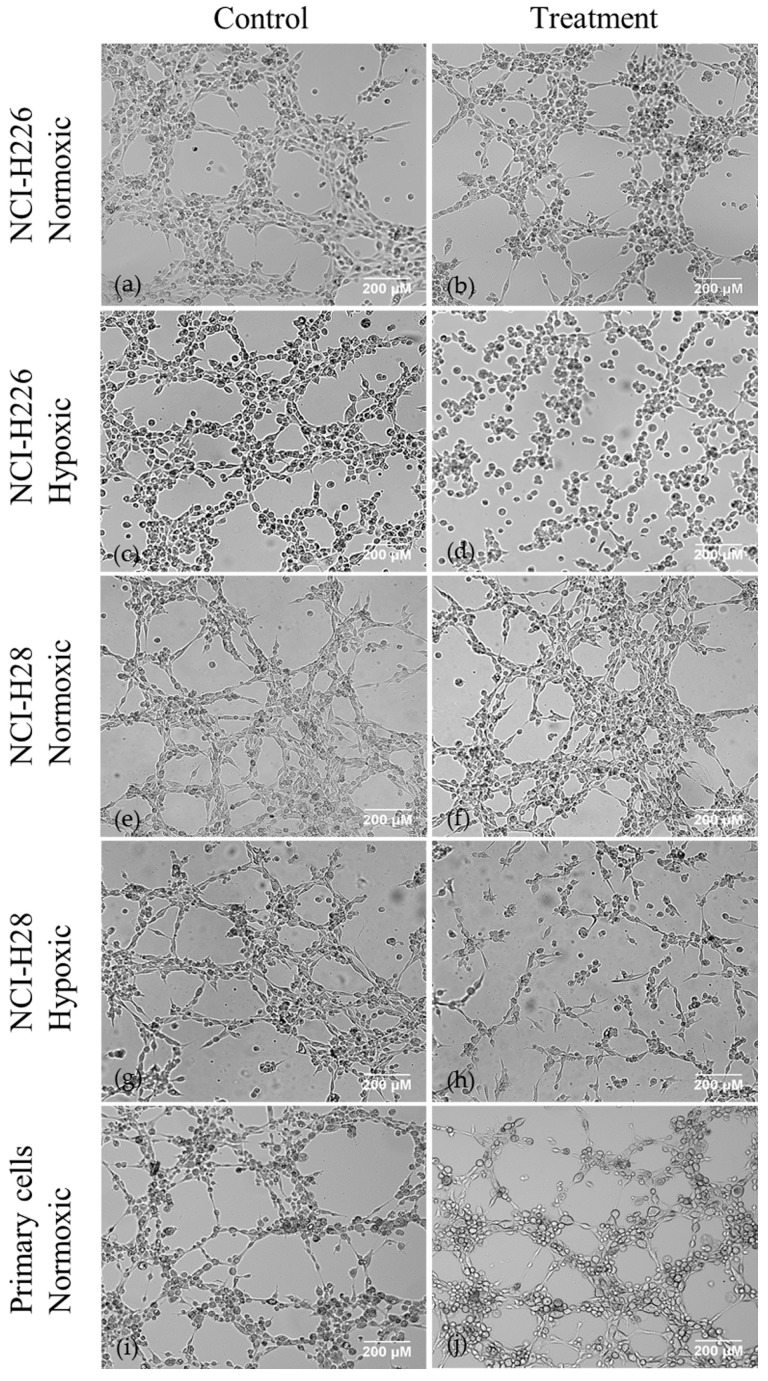
Vasculogenic mimicry (VM) assays on malignant mesothelioma (MM) cell lines and primary cells, Dimethyl sulfoxide (DMSO) control vs. AqB050 treatment. (**a**) control; and (**b**) treatment: AqB050 treatment (40 μM) does not inhibit VM in NCI-H226 cells under normoxic conditions; (**c**) control; and (**d**) treatment: AqB050 treatment (20 μM) significantly reduced branch count (*p* = 0.015) and loop count (*p* = 0.015) in NCI-H226 cells under hypoxic conditions; (**e**) control; and (**f**): treatment: AqB050 treatment (40 μM) does not inhibit VM in NCI-H28 cells under normoxic conditions; (**g**) control; and (**h**) treatment: AqB050 treatment (40 μM) significantly reduced branch count (*p* = 0.01) and loop count (*p* = 0.002) in NCI-H28 cells under hypoxic conditions; (**i**) control; and (**j**) treatment: AqB050 treatment (40 μM) does not inhibit VM in any of the MM primary cell lines tested under normoxic conditions. Analysed by Kruskal-Wallis test followed by Mann-Whitney U post hoc analysis.

Matrigel VM assays were performed using the VEGFA high- and low-MM cell lines NCI-H28 and NCI-H226 respectively (1878 pg/mL vs. 109 pg/mL seeded at 2.0 × 10^5^ cells/mL under normoxic conditions after 24 h), to observe potential differences in growth patterns depending on VEGFA status. The cells were treated with 20 μM and 40 μM of AqB050 under both normoxic (21% O_2_) and hypoxic (0.1% O_2_) conditions. Branch sites and loop counts were used to measure and compare VM [27,28]. Under normoxic conditions, AqB050 treatment did not inhibit VM in NCI-H226 or NCI-H28 cells compared with dimethyl sulfoxide (DMSO) control (Figure 1). Under hypoxic conditions, addition of 20 μM of AqB050 reduced branch count (*p* = 0.015) and loop count (*p* = 0.015) in NCI-H226 cells. In NCI-H28 cells, 40 μM of AqB050 was required to significantly reduce the branch count (*p* = 0.01) and the loop count (*p* = 0.002). AQP1 blockade did not inhibit VM in the three epithelioid primary cells tested under normoxia. Hypoxic experiments could not be performed in primary cells due to departmental safety regulations with regards to unknown mycoplasma status of primary samples. VEGF ELISAs were performed on cell supernatants to establish the VEGF status of each primary cell sample, despite showing no difference in VM under normoxic conditions. Of the primary cells tested, two patients had lower VEGFA protein levels (<2000 pg/mL) and one patient had high VEGFA levels (12,094 pg/mL) under normoxia.

**Figure 2:**
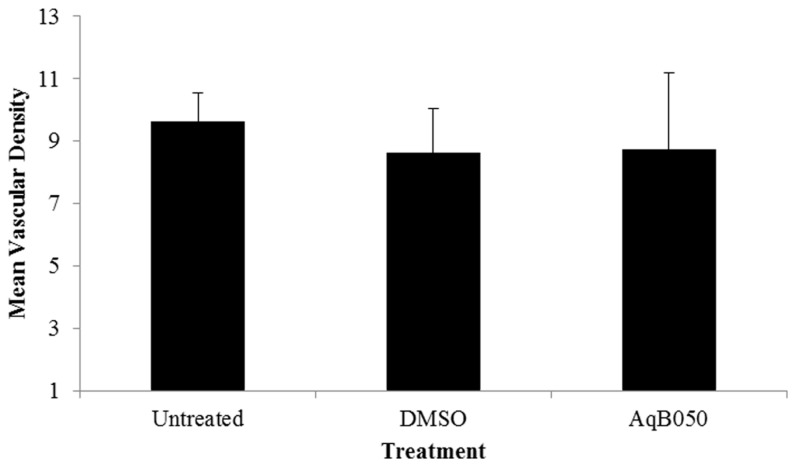
Mean microvessel counts in a MM xenograft model treated with AqB050. No difference in intratumoural vascular density per field was observed between untreated (range 8–12; *n* = 4), DMSO-treated (range 6–12; *n* = 4) and AqB050-treated (range 4–16; *n* = 4) groups in the in vivo mouse xenograft model. Analysed by one-way Analysis of variance (ANOVA), error bars: Standard error of mean (SEM).

In an in vivo xenograft model where NCI-H226 cells were injected subcutaneously into nude mice, AqB050 treatment (8.7 ± 4 counts/field) did not affect mean microvessel counts in vivo compared to DMSO-treated (8.6 ± 2.1) and untreated mice (9.6 ± 2.2) (Figure 2).

### 2.2. VEGFA Is Upregulated in NCI-H28 Cells When AQP1 Is Inhibited

**Figure 3:**
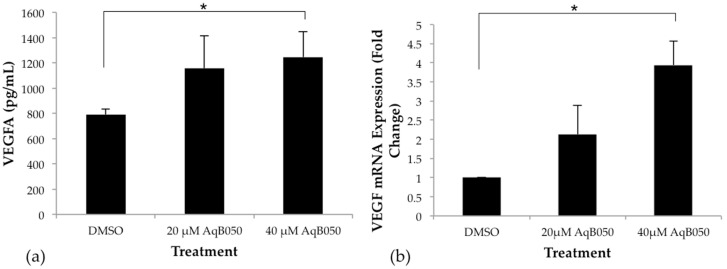
Treatment with 40 μM AqB050 alters (**a**) VEGFA protein secretion (*p* = 0.002) in NCI-H28 cells after 24 h; (**b**) confirmed by PCR where AqB050 treatment resulted in a 3.5–4.3 fold increase in VEGFA mRNA levels after 24 h (*p* = 0.002). Analysed by one-way ANOVA, followed by Tukey’s post-hoc analysis, error Bars: standard deviation (SD), *n* = 3; * indicates significance.

The addition of 40 μM AqB050 significantly increased VEGFA protein levels compared to DMSO control in NCI-H28 cells under normoxic conditions after 24 h incubation (*p* = 0.002, Figure 3a). Analysis of mRNA levels by qPCR showed that VEGFA mRNA expression increased 3.5–4.3 fold when treated with 40 μM AqB050 (*p* = 0.002), compared to DMSO control at 24 h (Figure 3b). In NCI-H226 cells, no significant difference was seen in VEGFA protein or mRNA levels with AqB050 treatment (data not shown).

### 2.3. Inhibition of VEGFA Alone Has No Effect on VM in NCI-H28 Cells and Combined Inhibition of AQP1 and VEGF Does Not Inhibit VM in NCI-H226 Cells

**Figure 4:**
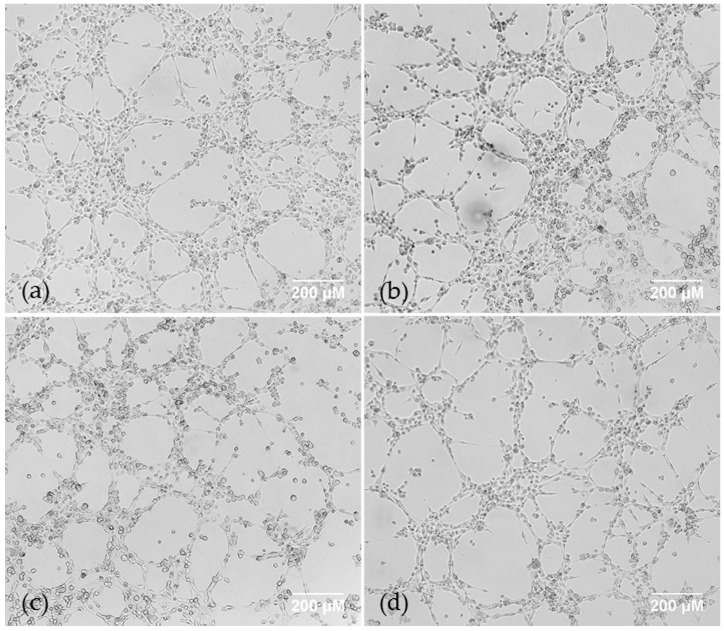
NCI-H226 cells in a Matrigel VM assay were treated with (**a**) DMSO control; (**b**) AQP1-specific siRNA alone; (**c**) bevacizumab alone; (**d**) a combination of AQP1-specific siRNA and bevacizumab. Treatment with 20 nM of AQP1 siRNA and 10 μg/mL of bevacizumab in combination or alone does not inhibit vasculogenic mimicry in NCI-H226 cells under normoxic conditions.

AQP1 inhibition using AqB050 was not sufficient to block VM under normoxia. To confirm this result, we used siRNA under the same conditions, alone and in combination with anti-VEGF antibody bevacizumab. Hypoxic conditions are known to upregulate VEGF, which to a degree negates potential effects of VEGF inhibition at clinically relevant concentrations, therefore normoxic conditions were used to further understand potential interaction between the two molecules. Treatment with bevacizumab at 10 μg/mL alone did not alter VM in either cell line compared to control human IgG. AQP1-specific siRNA, which had previously shown to functionally inhibit AQP1 in MM cells comparable to 40 μM AqB050 [20], was used to treat NCI-H226 cells. Cells were treated with 20 nM AQP1-specific siRNA ± 10 μg/mL bevacizumab, with negative control siRNA and human IgG used as controls. NCI-H226 cells treated with 20 nM AQP-1 specific siRNA and 10 μg/mL bevacizumab alone or in combination did not alter VM branch or loop counts compared to control (Figure 4).

## 3. Discussion

Retained AQP1 expression in MM surgical specimens has been associated with more favourable prognosis [24]. Although AQP1 is expressed by normal mesothelium, the expression of AQP1 is heterogeneous between tumours and it was initially hypothesised that retained AQP1 expression indicated greater differentiation. AQP1 was first described as a water channel, but has since been found to be involved in cell migration and proliferation [21,29,30]. It has also been observed that overexpression of AQP1 not only increases plasma cell membrane permeability by approximately 41% in colorectal cancer cells, and consequently promotes cell migration [31,32]. Blocking this channel, such as through the use of AqB050 in the current study, inhibits transmembrane water flux, and in turn this may inhibit dynamic shape changes in the cell that would usually favour a migratory phenotype that may aid in accommodating vessel formation. We have previously shown that inhibition of AQP1 by AqB050 or AQP1-specific siRNA decreases MM cell line motility, proliferation and colony formation [20]. The role of AQP1 in MM appears to be multifactorial. Due to its expression on vascular endothelial cells, where it may respond to hypoxic stimuli, and its role in cell motility, we proposed that there may be involvement of AQP1 in VM molecular pathways. As AQP1 has a documented role in proliferation of MM cells [20], to ensure primary focus on motility rather than proliferative activity, the VM experiments concluded after 6 h incubation. Additionally, cells can further migrate and ‘pile up’ after this time period, making analysis of vascular patterns difficult. Both NCI-H226 and NCI-H28 cell lines, which have very different baseline VEGFA levels, undergo VM under normoxic and hypoxic conditions within a 6 h period. It has been previously shown that AQP1 is expressed in NCI-H226 cells [20], and AQP1 protein expression in NCI-H28 cells was confirmed by immunohistochemistry before commencing in vitro experiments. Blockade of AQP1 with AqB050 inhibited VM under hypoxic conditions in both cell lines, but did not alter VM under normoxic conditions with either AqB050 or AQP1-specific siRNA. This may be attributed to the regulation of AQP1 by hypoxia-inducible factor-1α (HIF-1α). HIF-1 is a heterodimer comprised of an α and β subunit, the latter unaffected by oxygen levels [33,34]. Levels of HIF-1α are low in normoxia due to continuous proteasomal degradation, but are increased and stable under hypoxic conditions [34,35]. Expression of HIF-1α by immunohistochemistry has been observed in MM cells, but not in normal mesothelium [36]. Kaneko et al. studied the role of AQP1 in hypoxia-induced angiogenesis in human retinal vascular endothelial cells, finding a significant increase in AQP1 mRNA expression on exposure to hypoxia after 24 h, and a significant increase in AQP1 protein after 72 h [37]. They also reported a decrease in angiogenesis in cells transfected with AQP1-specific siRNA under hypoxic conditions in a Matrigel VM assay. This, in conjunction with our results, suggests that AQP1 may play a role in hypoxia-induced VM but is perhaps less involved in VM under normoxic conditions. It has been suggested that AQP1 may play a role in O_2_ homeostasis and transport, and thus inhibition of AQP1 may potentially imitate the effects of hypoxia and thus cause upregulation of hypoxia-sensitive molecules such as VEGF [38]. The presence of VEGF in normoxic or hypoxic conditions in human retinal vascular endothelial cells does not appear to have any direct effect on AQP1 expression [37]; however, Echevarría et al. reported increased VEGF mRNA expression with siRNA-modulated knockdown of AQP1 in a non-metastatic murine hemangioendothelioma cell line, comparable in vitro with microvascular endothelial cells [38], which is consistent with the results from NCI-H28 cells. Blockade of AQP1 may play a role in reducing VM in vivo due to the hypoxic tumour microenvironment, as was previously reported in a melanoma xenograft model [26]. To further investigate this, vascular density counts were performed on tumours from our mouse xenograft model, but no change was found in vascular density between AqB050 treatment and control groups. There was a large standard deviation within the treatment group, perhaps indicating that host factors may alter angiogenic pathways and response to the tumour in the presence of AqB050. An accurate depiction of the MM tumour microenvironment is also difficult to achieve in vivo with the previously mentioned typical heterogeneity of MM tumours and, therefore, variation in treatment response is expected. From the in vivo data, it is evident that AQP1 blockade may not be effective for reducing VM in vivo, perhaps due to individual variation and tumour cell heterogeneity.

It is clear that an abundance of pathways are implicated in MM pathogenesis, and specifically, angiogenesis, which stimulated our interest in the combined effect of AQP1 and VEGF inhibition. Increased VEGF levels in pleural effusions of MM patients are associated with adverse prognosis [39], and VEGF has been implicated in the development in malignant pleural effusion by increasing vascular permeability, not dissimilar to the function of AQP1 [40]. Despite this, the use of anti-VEGF agents in vivo in MM patients has yielded relatively unremarkable responses [5,6,7]. It was observed that NCI-H28 cells express up to 17 times more VEGF than NCI-H226 cells, and perhaps differences in VEGF expression between the two cell lines used in this study may help explain differences in behaviour. In vitro studies with MM cell lines have reported a reduction in cell viability and proliferation over 72 h with addition of VEGF neutralizing antibodies [41,42]. Additionally, treatment with 10 μg bevacizumab reduced endothelial cell proliferation and MM tumour microvessel density in an in vivo mouse xenograft model [42]. It was important for the purposes of measuring VM to stay within this time period to ensure any reductions in VM were not due to decreased cell viability. VEGFA blockade alone did not inhibit VM in either MM cell line. It was hypothesised that in NCI-H28 cells, which already produce high amounts of VEGF, more VEGF produced in response to AQP1 would saturate the available bevacizumab in solution, unless one would be prepared to go to clinically unachievable concentrations. This finding suggests that this cell line has a high dependency on VEGFA. This also suggests that the cells use high VEGF to co-opt native vessels more successfully and that VM is less likely to play a role clinically. However, it was possible that an effect may be seen on the VEGF-low NCI-H226 cells, where it was conceivable that both AQP1 and VEGF play a role. However, this did not occur, suggesting that neither the AQP1 nor the VEGF pathways are dominant in VM in normoxia, potentially explaining why anti-angiogenic strategies trialled in MM have been largely unsuccessful [4,5,6,7]. In clinical practice, tumour heterogeneity may be a potential issue for targeting specific molecules involved in VM. A lower concentration of AQP1 blocker was required to inhibit VM in NCI-H226 cells under hypoxic conditions, indicating that heterogeneity may account for differences in cell behaviour.

There is a clear association between VM, detected in patient samples by immunohistochemistry, and poor survival in hepatocellular carcinoma, gallbladder carcinoma, non-small cell lung cancer and osteosarcoma [43,44]. In other tumours, VM is associated with increased metastatic potential and increased tumour grade. An association between VM and patient survival has not yet been established in MM. In MM the number of reported cases was too low for definite prognostication but a majority of the reported cases were of sarcomatoid type which has a poor prognosis [15]. Testing primary cells from more patients would have been useful in interpretation of results; however, the availability of suitable primary cells is often limited. Many pleural effusions do not contain sufficient cells to successfully culture. We have previously seen that primary cells are quite heterogeneous even within the same subtype, exhibiting different behaviour between different time points when individuals were sampled. It is quite possible that different pathways are dominant in different individuals, as suggested by the different behaviour between NCI-H226 and NCI-H28 cell lines. Testing patient cells to determine VM status and subsequently targeting this process in VM-positive patients may have the potential to improve survival and quality of life.

In conclusion, we have established that inhibition of AQP1 suppresses VM under hypoxic conditions. It has long been suggested that pro-angiogenic VEGF is upregulated by HIF-1α, but it is evident that inhibition of AQP1 also results in upregulation VEGF in MM cells. Further investigation into the interaction between AQP1, VEGF and hypoxia may lead to a greater understanding of VM and angiogenic processes. Like angiogenesis, VM appears to be a result of a highly redundant and complex interaction of different molecules. It is conceivable that different cell lines and individual tumours will differ in their dependency on VM and/or angiogenesis for blood supply. Clinically, inhibition of tumour progression through limiting blood supply will likely require knockdown of multiple molecular pathways and will be dependent on individual tumour cell responsiveness.

## 4. Materials and Methods

### 4.1. Cell Culture

NCI-H226 and NCI-H28 cell lines were obtained from ATCC (Manassas, VA, USA). Human umbilical vein endothelial cells (HUVECs) were used as positive controls in VM assays, and were obtained from consenting donors. Primary cells were isolated from pleural effusion samples by centrifugation at 500× *g* for 5 min at 25 °C. The remaining cell pellet was cultured. This work was approved by the Southern Adelaide Clinical Human Research Ethics Committee (approval number 381.09, 20 April 2015). Cells were washed after 24 h with PBS to eliminate inflammatory cells and debris. Primary and MM cell lines were cultured in complete DMEM (10% foetal bovine serum, 50 U/mL penicillin, and 50 μg/mL streptomycin; Thermofisher, Waltham, MA, USA) and incubated at 37 °C in 5% CO_2_. HUVEC’s were cultured in in M199 media supplemented with 20% FCS, 50 U/mL penicillin, 50 μg/mL streptomycin, 1 μM sodium pyruvate, 1% *v*/*v* non-essential amino acids and 1% *v*/*v* GlutaMAX and incubated at 37 °C in 5% CO_2_. Hypoxia was achieved through the use of a Coy Laboratory Hypoxic workstation glove box (Coy Laboratory Products, Grass Lake, MI, USA). The chamber was kept at a controlled humidified temperature of 37 °C and was supplemented with 5% CO_2_, room air and N_2_ as required to maintain controlled O_2_ levels (0.1%). Cells were exposed to 0.1% O_2_ levels overnight before beginning experimentation. Hypoxia was confirmed by CaIX PCR.

### 4.2. AqB050 Aquaporin Blocker

The AQP1 specific pharmaceutical blocker, AqB050, was purchased from Prof Andrea Yool, University of Adelaide, South Australia, (US patent 7,906,555B2). The stock solution (40 mM) of AqB050 was prepared in DMSO and diluted to desired concentration for experiments. Previous investigations had confirmed that AqB050 was not cytotoxic to cells at working concentration [20].

### 4.3. siRNA Treatment

Cells were seeded in a 6-well plate at 1.4 × 10^5^ cells/well and allowed to adhere for 24 h before transfection with siRNA (Silencer Select siRNA, Life Technologies, Carlsbad, CA, USA) as per manufacturer’s instructions. Treatments of siRNA (AQP1, GAPDH and Negative Control) were complexed with Lipofectamine RNAiMAX (Thermofisher) in Opti-MEM (Gibco by Life Technologies, Carlsbad, CA, USA) and were administered at 20 nM. Cells were incubated for 72 h and then harvested for subsequent Matrigel VM assays and RNA extraction to confirm knockdown.

### 4.4. Matrigel VM Assay

To investigate the impact of AQP1 inhibition on vasculogenic mimicry, we treated the cells with AqB050, as well as AQP1-specific siRNA. We also used a combination of AQP1 siRNA (Cat # s1515, Life Technologies, Carlsbad, CA, USA) and bevacizumab (Avastin™, Genentech, USA) to investigate whether knockdown of AQP1 and VEGF would completely inhibit VM. Growth factor-reduced Matrigel (BD, North Ryde, NSW, Australia) was added to wells of a 15-well angiogenesis slides (Ibidi, Martinsried, Germany) and was allowed to polymerise for 30 min at 37 °C. AqB050 treatments (20 μM and 40 μM) and equivalent control DMSO (40 μM) (Sigma-Aldrich, St. Louis, MO, USA) were added to cells prior to seeding. For bevacizumab treatments, a concentration of 10 μg/mL of either bevacizumab or IgG isotype control was administered. Cell suspensions were then added to wells and were incubated for 6 h at 37 °C. Photos were taken one h and six h post incubation using AnalySIS getIT software (Olympus, Tokyo, Japan) and an F-view camera attached to the Olympus IX71 inverted fluorescence microscope (Olympus, Tokyo, Japan) with a 4× objective. Photos were saved as TIFF files and consecutive images stitched together using Photoshop CS5 software (Adobe, San Jose, CA, USA). Images were analysed for VM manually using ImageJ software (National Institute of Health, Rockville, MD, USA).

### 4.5. VEGFA ELISA

For comparison of NCI-H226 and NCI-H28 VEGFA secretion, cells were seeded at 1.6 × 10^5^ cells/mL in a 6-well plate and incubated for 24 h before supernatants were collected for ELISA analysis. For VEGFA quantification in the presence of AqB050, cells were seeded at 2.0 × 10^5^ cells/mL in a 6-well plate, and were incubated for 24 h before addition of 20 μM and 40 μM AqB050 or equivalent volume of DMSO. Cells were incubated for a further 24 h before supernatants were removed and cell pellets were harvested for RNA extraction. VEGFA protein was quantified using the DY293B Human VEGF DuoSet ELISA (R&D Systems, Minneapolis, MN, USA) according to manufacturer’s instructions. Sample concentrations were quantified by comparison to a standard curve.

### 4.6. Quantitative RT-PCR

Quantification of VEGFA and AQP1 in AqB050 treated cells and CaIX in hypoxic experiments was carried out using qRT-PCR. PPIA and B2M were used as housekeeping genes. RNA was extracted from cell pellets using the RNeasy RNA Extraction Kit (QIAGEN) with subsequent Turbo DNA-free (Ambion, ThermoFisher, Waltham, MA, USA) treatment to remove DNA contamination. The SuperScript III First-Strand Synthesis System (Invitrogen, ThermoFisher) was used for cDNA synthesis. TaqMan Gene Expression Assays (ThermoFisher) VEGFA (Hs00900055_m1), AQP1 (Hs01028916_m1), CAIX (Hs00154208_m), PPIA (Hs04194521_s1) and B2M (Hs00187842_m1) were used with TaqMan Fast Mastermix (ThermoFisher) in a StepOne Plus real-time PCR System (Applied Biosystems, Foster City, CA, USA). Reaction conditions were as follows: denaturation at 50 °C for 2 min, 95 °C for 20 s, then 40 cycles of 95 °C for 1 s, then 60 °C for 20 s.

### 4.7. Mouse Xenograft MM Model

We have previously described a subcutaneous xenograft model of MM [20]. NCI-H226 cells were injected subcutaneously into BALB/c nude mice. The three groups of animals examined included the untreated control group (*n* = 4), AqB050 treated group (20 μM, *n* = 4) and DMSO treated group (*n* = 4). Treatments were administered daily by intratumoural injection after tumours had reached approximately 100 mm^3^. Tumour size was calculated as previously described [20]. Animals were euthanised after tumours had reached 500 mm^3^ in size and xenografts were embedded and processed for histology. The work was approved by Flinders University and Southern Adelaide Local Health Network Animal Welfare Committee (805/12). Suitable tumour blocks with sufficient vascularisation were cut into 0.1 μM sections and stained with CD31 vascular marker. Sections were assessed by an RCPA-qualified Pathologist for intratumoural vascular density as previously described [45]. Three different fields were assessed at ×200 magnification for vessels with an identifiable lumen and counting vessels positively labelled with CD31 antibody.

### 4.8. Statistics

Statistical analysis was performed on IBM SPSS statistics version 2.22 (IBM, Armonk, NY, USA). Data which was normally distributed was analysed by one-way ANOVA with Tukey post-hoc analysis. Data which was not normally distributed was subjected to a non-parametric Kruskal-Wallis test followed by Mann-Whitney U post hoc analysis. A significant difference was accepted if *p* < 0.05.

## Abbreviations

MM	Malignant Mesothelioma
VM	Vasculogenic mimicry
VEGF	Vascular Endothelial Growth Factor
AQP1	Aquaporin 1
